# Market food diversity mitigates the effect of environment on women’s dietary diversity in the Agriculture to Nutrition (ATONU) study, Ethiopia

**DOI:** 10.1017/S136898001900051X

**Published:** 2019-04-17

**Authors:** Ramya Ambikapathi, Nilupa S Gunaratna, Isabel Madzorera, Simone Passarelli, Chelsey R Canavan, Ramadhani A Noor, Tshilidzi Madzivhandila, Simbarashe Sibanda, Semira Abdelmenan, Amare Worku Tadesse, Yemane Berhane, Lindiwe Majele Sibanda, Wafaie W Fawzi

**Affiliations:** 1 Department of Global Health and Population, Harvard T. H. Chan School of Public Health, Boston, MA, USA; 2 Department of Nutrition Science, Purdue University, Stone Hall G1G, 700 West State Street, West Lafayette, IN 47907, USA; 3 Public Health Graduate Program, Purdue University, West Lafayette, IN, USA; 4 Food, Agriculture and Natural Resources Policy Analysis Network, Pretoria, South Africa; 5 Addis Continental Institute of Public Health, Addis Ababa, Ethiopia

**Keywords:** Agriculture, Nutrition, Market food diversity, Farm diversity, Livestock diversity

## Abstract

**Objective:**

In Ethiopia, women’s dietary diversity is low, primarily due to poor food availability and access, both at home and market level. The present study aimed to describe market access using a new definition called market food diversity (MFD) and estimate the impact of MFD, crop and livestock diversity on dietary diversity among women enrolled in the Agriculture to Nutrition (ATONU) trial.

**Design:**

Baseline cross-sectional data collected from November 2016 to January 2017 were used for the analysis. Availability of foods in markets was assessed at the village level and categorized into nine food groups similar to the dietary diversity index for women. Bivariate and multivariate mixed-effects regression analyses were conducted, adjusted for clustering at the village level.

**Setting:**

Chicken-producing farmers in rural Ethiopia.

**Participants:**

Women (*n* 2117) aged 15–49 years.

**results:**

Overall, less than 6% of women met the minimum dietary diversity (≥5 food groups) and the most commonly consumed food groups were staples and legumes. Median MFD was 4 food groups (interquartile range: 2–8). Multivariate models indicated that women’s dietary diversity differed by livestock diversity, food crop diversity and agroecology, with significant interaction effects between agroecology and MFD.

**conclusions:**

Women’s dietary diversity is poor in Ethiopia. Local markets are variable in food availability across seasons and agroecological zones. The MFD indicator captures this variability, and women who have access to higher MFD in the highland agroecological zone have better dietary diversity. Thus, MFD has the potential to mitigate the effects of environment on women’s dietary diversity.

In rural areas of the global South, there are high rates of undernutrition and poor dietary quality, primarily due to low food availability and access, both at household and market levels^(1,2)^. In an effort to address poor access to nutritious foods as the source of undernutrition, interventions have largely focused on promoting home-based production^(3)^. In Ethiopia, one-quarter of rural women of reproductive age are undernourished^(4)^. Recent studies have found that improving home production diversity is positively associated with women’s dietary diversity, but these effects are small and plateau at a certain threshold, suggesting that a multitude of other factors may affect dietary diversity^(2,5–7)^.

These factors include market access (participation, size, physical and economic access), technology, livestock ownership, women’s role in decision making, and demographic factors such as wealth and education^(8)^.

In addition to these factors, variability in agroecological landscape poses an inherent and substantial limitation on land use, consequently affecting the production and yield of both livestock and crops, and, ultimately, the dietary intake of the population. To overcome agroecological limitations, studies have highlighted issues including access to and integration of local markets, informal seed exchange, increased communal connectivity, and cultivation at different altitudes to reduce risk and crop loss^(9,10)^. Access to markets presents a particularly complex and inconsistent relationship. In one study in Ethiopia, an interaction between on-farm food crop diversity and market access on dietary diversity of children was observed^(10)^. In another nationally representative Ethiopian study, increased market distance had a negative effect on household dietary diversity, while no interaction effects between production and market were observed regarding household dietary diversity^(2)^. Another study from northern Ethiopia concluded that diets were generally more diverse among children who resided closer to the market only in the post-harvest season (October to April)^(11)^. The same authors noted that the consumption of animal-source foods was affected by market access, particularly in the harvest season.

Inconsistency in the relationship between market access and rural diets comes from using different definitions of market access, different target groups (children, households), varying definitions of the outcomes (utilizing seven *v*. twelve food groups), differences in methods in evaluating the outcome (24 h *v*. 7 d recall) and study designs^(8)^. The present paper adds to the growing literature on the linkages between market, agriculture and dietary intake; in particular, we introduce a new definition of a market characteristic that captures the dynamic nature of food availability.

In the current analysis, we examine determinants both at home (crop diversity, livestock diversity) and in the market (market food diversity) to estimate the relative and synergistic effects of these elements on the dietary diversity of women in Ethiopia using baseline data collected from the Agriculture to Nutrition (ATONU) trial in Ethiopia. We utilize a newer definition of a market characteristic called ‘market food diversity’, in accordance with the indicator of Minimum Dietary Diversity for Women^(12)^. We describe market food diversity as the availability of foods and food groups across seasons and agroecological zones using the information collected from traders and developmental agents, and from physical visits to the markets, which is in contrast to the previously used method, where Bellon et al. used purchased food items from a 7 d recall of food consumption as a measure of market diversity^(5)^. Specifically, we hypothesize that after adjusting for household wealth and demographic characteristics, food crop, livestock and market food diversity will be positively associated with dietary diversity among women.

## methods

The ATONU study is a cluster-randomized trial in Ethiopia that examines the impact of chickens and nutrition-sensitive agriculture interventions on women’s and children’s dietary diversity and nutritional status (Clinicaltrials. gov identifier: NCT03152227). The trial consists of three waves of data collection in four regions of Ethiopia (Amhara; Oromia; Southern Nations, Nationalities and Peoples’ Region (SNNPR); Tigray) within twenty woredas. Clusters were defined at the kebele (village) level. Baseline data collection was conducted from November 2016 to January 2017, where 2407 households were screened. Among these, 290 households did not meet the eligibility criteria for different reasons: eight households did not consent to participate, fifty-five did not raise chickens in the past two years, eleven had more than fifty chickens, 182 did not have a woman of reproductive age between 15 and 49 years old, seven did not intend to stay in the area and twenty-eight had missing forms.

### Key outcome variables

Food consumption was assessed using a food list questionnaire that queried the respondents on seventy foods items consumed in the preceding day (24 h) and 7 d. Women’s diet diversity score was calculated as the number of food groups out of ten, consumed in the preceding day based on a standardized indicator definition, Minimum Dietary Diversity for Women (MDD-W)^(12)^. Overall, (i) staples included thirteen foods (teff, maize, rice, wheat, sorghum, barley, pasta, other grains, enset, white sweet potato, white yam, white cassava, other white starchy staple); (ii) pulses included seven foods (common beans, chickpeas, lentils, peas, cowpeas, bambara, other bean/ peas); (iii) nuts/seeds included four foods (sunflower seeds, sesame seeds, groundnuts, other nuts); (iv) dairy included four foods (milk, cheese, yoghurt, other milk products); (v) flesh foods included nine foods (liver, organ meat, beef, goat, chicken, other poultry meat, lamb, other flesh protein, fish/dried fish); (vi) eggs included two foods (chicken eggs, other eggs); (vii) dark green leafy vegetables included six foods (kale, spinach, morenga, cassava leaves, sweet potato leaves, other dark green leafy greens); (viii) vitamin A-rich fruits and vegetables included nine foods (pumpkin, carrots, orange-fleshed sweet potatoes, bereket, other vitamin A-rich vegetables, ripe mango, ripe papaya, other juices from orange-flesh fruits, other orange-flesh fruits); (ix) other vegetables included seven foods (tomato, onion, eggplant, green pepper, cucumber, cabbage, other local vegetables); and (x) other fruits included eight foods (ripe banana, guava, pineapple, apple, orange, beles (prickly pear), other local/wild fruits, 100% fruit juice from fruits).

### Independent variables

There are three main key exposures of interest: (i) food crop diversity; (ii) market food diversity; and (iii) livestock diversity. For food crop diversity, seasonal estimates (the main Meher season, harvested between September to February, and the minor Belg season, harvested between June to August) were calculated based on crop surveys collected from the head of the household. Food crop diversity is determined by categorizing fifty-two crops into seven food groups^(12)^, not including the three food groups that involve animal-source foods. In addition, a cash crop indicator was created if the farmers grew any of the following crops: chat, sugarcane, coffee or tobacco. We included 121 households in the analysis that did not have any food crop diversity in either of the seasons. Among these 121 households, 68.6 % households grew cash crops.

For market food diversity, vendors and developmental agents from each village were asked about monthly food availability and food prices (per specified unit, purchase price, selling price) in their local market. There were two such key informants for each village. Based on availability of twenty-five foods, nine food groups were created to form ‘market food diversity’ per month per village (not including the nuts and seeds food group as these foods were not available in any market) in accordance with the MDD-W indicator^(12)^. The twenty-five foods were: maize, wheat, sorghum, barley, common beans, chickpeas, lentils, peas, dark green leafy vegetables, pumpkin, carrot, mango, papaya, green pepper, banana, orange, milk, yoghurt, organ meat, beef, live chickens, local chicken eggs, exotic chicken eggs, cooking oil and butter (oil and butter were not included in market food diversity definition). More information on the survey tool can be found at www.fanrpan.org ^(13)^. For the purposes of temporality, we retained market food diversity as a continuous indicator for the month of November 2016 in relation to the main outcome, also collected in November 2016. For comparison purposes, we developed a binary indicator of market food diversity, defined as ‘low market food diversity’ if markets had four or fewer food groups. We conducted sensitivity analyses using five food groups and did not find significant differences between the two definitions among our key exposures of interest.

Lastly, livestock diversity was estimated by three different definitions: (i) current count of different species at the household level; (ii) tropical livestock units (TLU, a categorization based on grazing land used by an animal) based on current livestock owned^(14)^; and (iii) specific indicators of current ownership of cattle, goats/sheep or chickens.

Woman-level factors adjusted for in the analyses included age and education. Household-level factors included wealth quintiles, household size, elevation (metres), access to improved sanitation and access to protected water sources. Wealth quintiles included the first component from a principal component analysis, which included household assets, having an improved roof, wall or floor, having electricity and the size of land ownership, and explained 9.5 % of the overall variance.

### Statistical analysis

Bivariate models with village-level random effects were compared with multivariate models. Multivariate mixed-effects models were estimated with the village as the random intercept to adjust for clustering. Treatment, region, agroecological zone and other covariates were treated as fixed effects. There were four administrative regions sampled in the ATONU trial (see online supplementary material, Supplemental Table 1).

**Table 1:** Demographic information of the participating households (*n* 2117); Agriculture to Nutrition (ATONU) study, rural Ethiopia, November 2016–January 2017

Characteristic	Median, Q1–Q3 or %
Women’s age (years), median	35
Q1–Q3	28–40
Household size (n 2116), median	6
Q1–Q3	5–8
Women-headed household (%)	12.4
Women’s education (%)	
No schooling (˜1 year)	58.0
Primary 1 (2–5 years)	194
Primary 2 (6–9 years)	140
Higher education (secondary 1, 2, university)	5.7
Other (religious, adult literacy)	2.9
Region (%)	
Amhara	24.9
Oromia	30.9
SNNPR	24.9
Tigray	19.3
Agroecological zone (%)	
Highland	18.5
Lowland	24.7
Midland	56.8
Access to electricity (%)	23.5
Access to improved sanitation (n 2112; %)	30.5
Access to improved water (*n* 2100; %)	83.4
Major type of wall (%)	
Rudimentary wall	85.4
Type of roof (%)	
Finished roof	75.9
Rudimentary roof (palm, grass, leaves)	21.9
Other	2.2
Size of land owned (timads)*, median	3
Q1–Q3	2–6
Farm size (timads)*, median	4
Q1–Q3	2–7

Q1, quartile 1; Q3, quartile 3; SNNPR, Southern Nations, Nationalities and Peoples' Region.

*4 timads = 1 ha.

### Interaction term

In Ethiopia, agroecological zones are defined by rainfall, elevation and length of growing season, and were characterized intro three groups: lowland, midland and highland. In terms of elevation, lowlands are typically below 1500 m, midlands are between 1500 and 2300 m, and highland areas have an altitude greater than 2300 m^(15)^. In terms of rainfall, lowland zones receive less than 800 mm/ year, midland zones about 800–1200 mm/year and highland zones greater than 1200 mm/year. Overall, highland zones have the highest number of growing days at 240 d, midland zones have about 120–240 d and lowlands have less than 120 d (see online supplementary material, see Supplemental Table 2)^(15)^. In addition to agroecological zones, market food diversity was tested as an interaction term with food crop diversity because of the direct relationship to the length of the growing season (which is also affected by agroecological zone).

**Table 2:** , crop, livestock and market food diversity in the sample population (*n* 2117), overall and by agroecological zone; Agriculture to Nutrition (ATONU) study, rural Ethiopia, November 2016–January 2017

Diversity indicator	All		Lowland		Midland		Highland	
	Median or %	Q1–Q3	Median or %	Q1–Q3	Median or %	Q1–Q3	Median or %	Q1–Q3
Women’s dietary diversity in the previous 24 h	3	2–3	3	2–4	3	2–3	2	2–3
% meeting minimum women’s dietary diversity (five or more food groups)	5.6		9.0		5.1		2.0	
% of women fasting in the previous 24 h	13.1		8.8		13.0		19.0	
Women’s dietary diversity from 7 d recall	4	3–5	4	3–5	4	3–5	3	2–4
Market food diversity	4	2–8	3	1–8	5	2–8	4	2–7
% with low market food diversity*	51. 3		61. 4		45.7		55.1	
Food crop diversity (Meher) †	1	1–2	1	1–2	1	1–2	1	1–2
% growing crops in Meher	93.8		97.1		93.0		91.6	
Food crop diversity (Belg) †	1	1–2	1	1–1	1	1–2	1	1–2
% growing crops in *Belg*	27.4		9.6		26.4		54.6	
% growing cash crops (coffee, chat or sugarcane)	20.0		3.6		32.5		3.3	
Livestock diversity (species)	3	2–4	3	2–4	3	2–4	3	3–4
% of households that own any livestock (%)	99.9		100.0		99.8		99.7	
Tropical livestock units	2.1	1–3	2.7	1–4	1.9	1–3	1.9	1–3
Number of cattle owned per household‡	3	2–4	4	2–4	3	2–4	3	2–4
% of households that own cattle	86.1		87.6		83.9		91.3	
Number of goats/sheep owned per household‡	3	2–6	4	3–9	3	2–6	3	2–4
% of households that own goats/sheep	54.5		51.2		53.1		63.3	
Number of chickens owned per household‡	6	3–15	6	3–19	7	3–16	4	2–19
% of households that own chickens	87.7		87.4		88.4		85.7	

Q1, quartile 1; Q3, quartile 3.

*Low market food diversity defined as less than five food groups; refers to November 2016 market food diversity.

†Only out of seven food groups because it does not include dairy, eggs or meat food groups; values represent among those who planted crops. ‡Among those who own specific livestock.

## results

The demographics and household characteristics of households enrolled in the ATONU trial at baseline are shown in Table 1. On average, households had six members and were predominantly male-headed (88 %). A typical household owned 3 timads (0.75 ha) of land. Most of the households had access to improved water but less than one-third had access to improved sanitation or electricity. Over half of households were found in the midland agroecological zone. Although there were no differences in women’s education and improved water access across regions, there were differences in terms of access to sanitation and electricity (see online supplementary material, Supplemental Table 1).

Median age for women was 35 years and over half had no formal education. Less than 6 % of the women met the recommended minimum dietary diversity of at least five food groups consumed in a day (Table 2). These varied by agroecological zone and region. Oromia and lowland agroecological zones had the highest percentage of women meeting the minimum recommended dietary diversity, with 11.8 and 9.0 %, respectively. Tigray and the highland agroecological zone had the lowest percentage of women meeting the recommendation at 1.5 and 2.0 %, respectively. The median number of food groups consumed by women in the previous day was three, which included grains (>99 %), other vegetables (72 %) and legumes (53 %), and this was consistent across agroecological zones. From the 7 d recall, women reported consuming four food groups; three were similar to the 24 h recall, with an addition of dairy consumption (37 %). Thirteen per cent of the women reported that they were fasting (consumption of vegan food in observance of religious activities, usually on Wednesdays and Fridays) on the day food recall data were collected; this was slightly lower in the lowland zone, with less than 9 % of women reporting fasting days. While fasting increased the consumption of legumes among women, it was not significantly associated with dietary diversity in the bivariate or multivariate models, and accordingly was not included in the final adjusted model. Day of the week, however, influenced dietary diversity among women, particularly on Fridays (data were collected on Saturdays). On average, dietary diversity score on Fridays was 0.30 lower compared with Mondays (*P* = 0.001) in the bivariate model but was not significant in the multivariate models. Less than 5% of the women described the day of the dietary recall as a holiday and this was not associated with the dietary diversity score.

There was high livestock ownership in this population, with 50 % of households owning at least three animal species. The most commonly owned animals were cattle, chicken and small ruminants (goats or sheep). A typical household owned three cows/bulls, six chickens and three small ruminants. Households in the lowland and midland zones had higher numbers of chickens compared with households in the highland agroecological zone. About 75 % of households grew only one to two food crops (groups) during both Meher and Belg seasons, and this was consistent across regions. However, in the Belg season, only one-third of households grew any food crops, and these were mostly grains. In the entire sample, one-fifth of households grew cash crops (coffee, chat, sugarcane) and these households were mostly located in the midland agroecological zone.

**Fig. 1:**
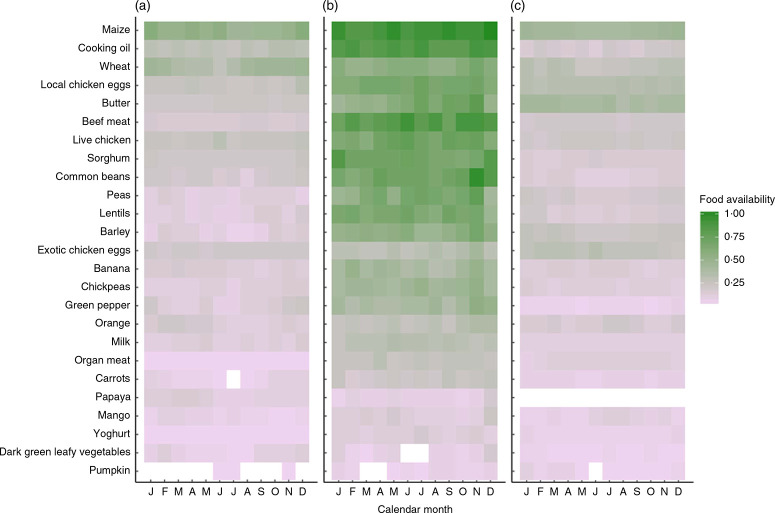
Proportion reporting market food availability (as indicated by two key informants in each village) by agroecological zone: (a) lowland, (b) midland and (c) highland; Agriculture to Nutrition (ATONU) study, rural Ethiopia, November 2016–January 2017. Green colour or density of 1.0 denotes that all markets assessed in the particular zone and month have availability of the selected foods, while light purple and grey (density of 0.0) denotes very low availability. Food items are ordered by overall availability, where maize was nearly universally available in markets and pumpkin was the least available. Blanks represent no availability; for example, carrots are not found in the month of July among markets in the lowland agroecological zone

Market food diversity in November 2016 was evenly distributed across the nine food groups, and half of households had access to a market with at least five food groups. The most common food groups found at the market were: grains (93 %), pulses (76 %), meat (66 %), eggs (63 %) and fruits (46 %). Figure 1 illustrates the availability of each of the twenty-five foods at the markets found in each agroecological zone across 12 months. Overall, Fig. 1 shows that there is poor access to vitamin A-rich foods such as carrots, papaya, mango, dark green leafy vegetables and pumpkin across all three agroecological zones and in both seasons. Foods like wheat, eggs, butter and barley show strong seasonality. The midland agroecological zone appears to have the highest diversity of food, with a median market food diversity of five food groups; the lowland agroecological zone has a median of three food groups; and the highland zone has a median of four food groups.

**Fig. 2:**
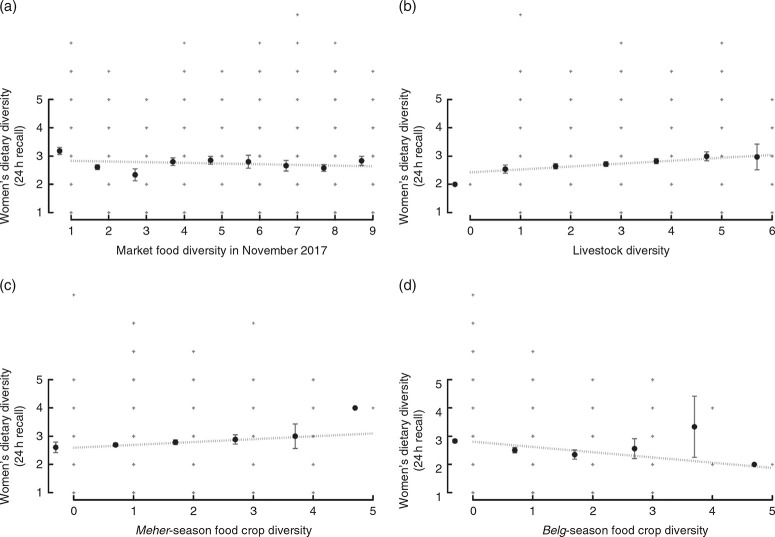
Relationship between women’s dietary diversity in the previous 24 h and (a) market food diversity in November 2017, (b) livestock diversity, (c) food crop diversity in *Meher* season and (d) food crop diversity in Belg season; Agriculture to Nutrition (ATONU) study, rural Ethiopia, November 2016–January 2017. Values are means (•), with their standard errors represented by vertical bars; + represent the range of dietary diversity scores. Slope and *P* value from bivariate models adjusted for kebele: (a) slope = –0.02, *P* = 0.286; (b) slope = 0.10, *P*< 0.001; (c) slope = 0.13, *P*< 0.001; (d) slope = 0.05, *P* = 0.179

The relationships between women’s dietary diversity in the previous 24 h and market, livestock and crop diversity (in two seasons) are shown in Fig. 2. Livestock diversity and Meher-season food crop diversity have positive and linear associations with women’s dietary diversity. Market food diversity appears to have a curvilinear relationship with women’s dietary diversity, where women’s dietary diversity plateaus past three market food groups. Belg-season food crop diversity appears to show a biconvex relationship with women’s dietary diversity, noting that very few households grow four or five different food crops.

**Fig. 3:**
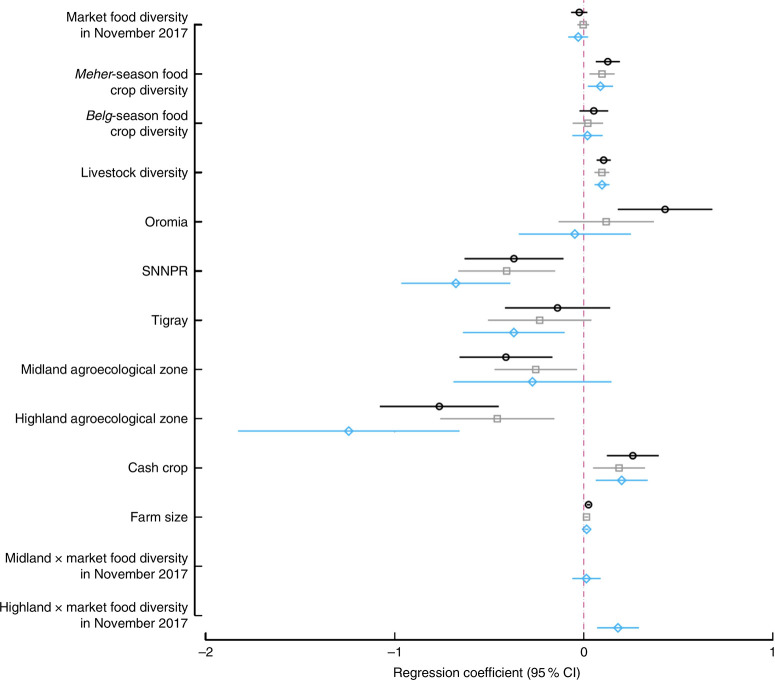
Regression results from models examining the effect of market food, food crop and livestock diversity, region and agroecological zone on women’s dietary diversity; Agriculture to Nutrition (ATONU) study, rural Ethiopia, November 2016–January 2017. Values are regression coefficients, with their 95% CI represented by horizontal bars, from bivariate models (○), multivariate models without interaction terms (□) and multivariate models with interaction terms (◊). Multivariate models adjusted for region, education, women’s education, wealth quintiles, woman’s age, age of the household head, household size, access to improved water and sanitation, and kebele-*level* clustering (SNNPR, Southern Nations, Nationalities and Peoples’ Region). Other coefficients are presented in the online supplementary material, Supplemental Table 3

**Fig. 4:**
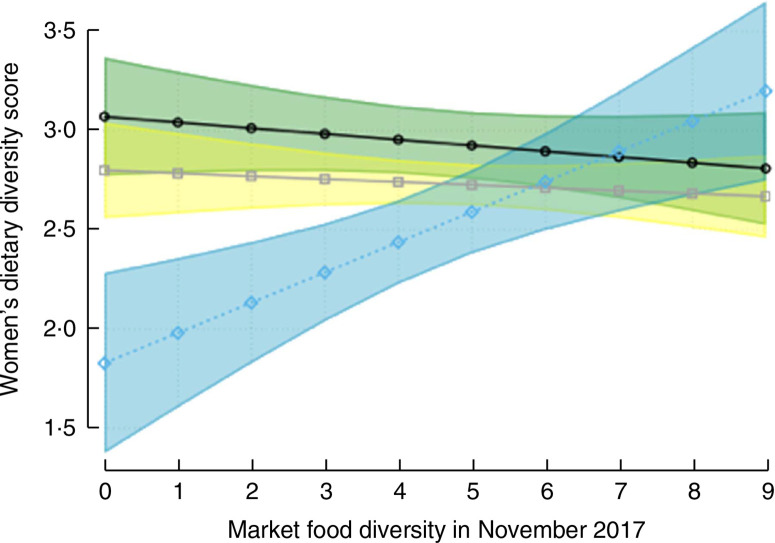
women’s dietary diversity score, with 95 % confidence interval represented by shaded band, from the adjusted multivariate model that includes interaction between agroecology (□, □, lowland;F020 □, □, midland; □, □, highland) and market food diversity; Agriculture to Nutrition (ATONU) study, rural Ethiopia, November 2016–January 2017. Highland agroecological zone × market food diversity has estimated slope = 0.18 and P = 0.001

Results from bivariate, multivariate (no interaction) and multivariate with interaction models are summarized in Fig. 3. In the bivariate models, Meher-season food crop diversity, livestock diversity, TLU, cultivation of cash crops, farm size, belonging to the highest wealth quintile, living in Oromia region (compared with Amhara) and having secondary school education had significant and positive associations with women’s dietary diversity. Meanwhile, access to improved sanitation, woman’s age and living in the midland and highland zones had significant and negative associations with women’s dietary diversity. We did not find any baseline differences by treatment arm, thus removed the treatment in the final models. In the multivariate model without interaction, cultivation of one additional food group during the Meher season was associated with an increased women’s dietary diversity of 0.09 food groups (*P* = 0.004), which was similar in magnitude and direction to the relationship of adding one additional livestock species to the household (*P* < 0.001). Producing cash crops was associated with a 0.20-higher dietary diversity (*P* = 0.004), while residing in midland and highland agroecological zones was associated with a significantly lower women’s dietary diversity of 0.26–0.49 compared with living in the lowlands. There was a similar effect size when the models were run with market food diversity as a categorical rather than continuous variable, or when TLU or livestock ownership was added instead of livestock diversity. Market food diversity did not have any association with women’s dietary diversity in the bivariate or the multivariate no-interaction model. When the interaction term of agroecological zone and market food diversity was tested, the interaction term for highland agroecological zone was positive and significant (*P* = 0.001). Predicted women’s dietary diversity scores from the interaction model are shown in Fig. 4. Figure 4 illustrates that increases in market food diversity in the highland agroecological zone increased the dietary diversity of women, where the women’s dietary diversity was the lowest overall (coefficients from the bivariate and multivariate models are shown in the online supplementary material, Supplemental Table 3). In other words, women who have access to diverse markets in the highland agroecological zone have better dietary diversity.

## discussion

In rural Ethiopia, there are high rates of undernutrition among women of reproductive age. In order to address food access and availability issues in rural areas, interventions have focused on home production of livestock and crops. These interventions have differential effects on women’s dietary diversity based on access to markets. In the present study, we used a new definition of market characteristics called market food diversity to evaluate the impact of household-level factors, such as food crop diversity and livestock diversity, on women’s dietary diversity. Overall, 95 % of women did not meet the recommendations for MDD-W. Over half of households had a local market that had at least five food groups, and the food groups available in these markets varied by agroecological zone and season. There was low availability of vitamin A-rich produce across the four regions of Ethiopia and in both seasons. Regression model results showed that food crop diversity in the main *Meher* season was positively associated with women’s dietary diversity. Livestock diversity (assessed as species diversity) was also positively associated with women’s dietary diversity and this effect was consistent regardless of the three definitions used in the model. Market food diversity in the preceding month mitigates the effects of environment among women living in the highland agroecological zone. Growing cash crops such as sugarcane, chat and coffee had a positive and stronger relationship with women’s dietary diversity, compared with food crop diversity. Higher women’s education showed a marginally positive association with dietary diversity (P < 0.10); while residing in SNNPR or the midland or highland agroecological zone was associated with lower women’s dietary diversity.

We did not find a significant association of women’s dietary diversity with weekday fasting, woman’s age, access to improved water or elevation of the household. These results were expected. First, women’s education and access to improved water were fairly homogeneous in this sample population. There was a low prevalence of women fasting on the day of dietary recall, therefore this variable was unlikely to influence our model estimates. Elevation did not impact women’s dietary diversity in the current study because any impact of elevation on household production was already controlled for agroecological zone and farm size.

For the present analysis, we focused on describing markets and household food availability across agroecological zone because studies in the past have indicated: (i) small, positive effects of food crop diversity on dietary diversity of household members; and (ii) that market food availability for the household has an impact on the relationship between food crop and dietary diversity, but only in certain regions of the world. In other words, households’ food crop diversity among those located closer to the market has a lower impact on dietary diversity, while in remote locations, household food crop diversity has a higher effect on dietary diversity^(2,6,8,16)^. Our results were consistent with the literature, where we found a small and positive effect of food crop diversity in the main season on women’s dietary diversity. We posit that the relationship between *Meher*-season food crop diversity and women’s dietary diversity is manifested primarily through the income pathway (purchasing power capacity) and these effects are small because of temporality. We do not see a strong relationship with *Belg* season and women’s dietary diversity, likely due to low prevalence of food crops grown in this season. We do see that growing cash crops (in either *Belg* or *Meher* season) positively influences women’s dietary diversity, again through the income pathway.

We found interaction effects when market food diversity was used as a binary indicator (high v. low market food diversity) among those who are growing a higher diversity of food crops; however, this effect became non-significant when there was a market food diversity interaction with agroecological zone. There are several possible explanations for these observed associations: (i) market food diversity as a continuous indicator captures the dynamic measure of food availability and to some extent latently captures access, and this resolution is lost when using a binary indicator; (ii) the interaction term with agroecological zone attenuates other interactions (especially those related to the environment); and (iii) the temporal lag between food crop diversity and women’s dietary diversity is large, such that interaction effects between food crop and market on women’s dietary diversity might be attenuated. Further research on water insecurity by agroecological zone may shed light on influences on food crop and women’s dietary diversity^(17)^.

Many of these past studies are focused on household or child’s dietary diversity rather than women’s dietary diversity, so the effects may vary across household members. In Ethiopia, analysis of a nationally representative sample found that market distance impacted household dietary diversity negatively using a 7 d food recall but did not find any interaction between market distance and food production diversity^(2)^. This was inconsistent with the analyses conducted by the International Food Policy Research Institute in the northern region of Ethiopia for children’s dietary diversity^(10,11,18)^. In particular, these studies highlight several important factors that could improve children’s dietary diversity. First, seasonality and agroecological zone play a role in market food diversity. Second, the components of diet that were altered due to market access might be limited to one or two food groups (dairy, in the case of the analysis conducted in Tigray region in Ethiopia). Finally, a child’s caregiver’s nutrition knowledge substantially improved dietary diversity, but only in areas with good market access^(18)^. It is possible that improving men’s and women’s nutrition knowledge may benefit dietary diversity of all members of the household. In our present analysis conducted in the ATONU trial, we find that the effect of agroecological zone on women’s dietary diversity was mitigated by having higher market food diversity, keeping in mind that these effects may not translate to children’s dietary outcomes because children may already have nutritional buffering (i.e. mothers prioritizing food allocation for children over themselves). It is also important to note that these studies have measured market access through distance or participation, while in the present analysis we do not have information on these previously used indicators.

The present analysis has several limitations. First, the study did not gather information on distance from the household to the market or frequency of market participation. Further information on these aspects can shed light on engagement of the households with the market. Second, investigators recognize that markets are not randomly distributed across regions, which may have an impact on the directionality of associations. Third, information on nuts and seeds was not collected in the market survey, hence we did not use it in the creation of the market food diversity indicator. Additionally, there was a larger sample size in the midland agroecological zone compared with the other two zones. Lastly, the analysis is cross-sectional so any associations found in the present study do not imply causality.

There are also several strengths to the current study. The ATONU trial had a study population from a wide geographic range (four major regions of Ethiopia). Furthermore, the ATONU trial collected individual, household and community (village) level variables that provided a broader view of market food availability and diversity in Ethiopia. We found consistent results when we used multiple definitions of main independent variables; for example, market food diversity as a continuous and categorical variable, and livestock diversity as TLU, count and species diversity. Finally, we were cognizant of temporality in terms of interactions between market food diversity on women’s dietary diversity, thus we retained the market food diversity in the previous month as the major exposure of interest. However, we recognize that crop production in the previous season affects women’s diversity through the income pathway (from selling in the market) rather than direct consumption of food. As illustrated in Fig. 1, it is evident that market food diversity varies seasonally and by agroecological zone. Currently, the most commonly used indicator of market access is distance to markets, which does not vary by season.

Aligning the definition of market food availability with the women’s dietary diversity indicator and applying it to local markets provides a relevant and dynamic measure of locally available nutritious foods, which is informative for tailoring interventions and targeting programmatic efforts. The association of market food diversity mitigating the effects of growing season is promising, and further longitudinal analyses should be conducted to evaluate the strength and directionality of this effect.

## Supplementary materials

To view supplementary material for this article, please visit https://doi.org/10.1017/S136898001900051X
