# Supplementary material for: Market food diversity mitigates the effect of environment on women’s dietary diversity in the Agriculture to Nutrition (ATONU) study, Ethiopia
Source: Public Health Nutr. 2019 Apr 17;22(11):2110–9. doi: 10.1017/S136898001900051X (PMC7745109; doi:10.1017/S136898001900051X)
Supplement: Supplementary file 1 [file S136898001900051Xsup001.docx]

**Supplemental Table 1: Demographic information, market diversity, food crop diversity, market food diversity by regions.**

|  | Amhara | Oromia | Southern Nations, Nationalities, and Peoples' Region | Tigray |
| --- | --- | --- | --- | --- |
| Sample size | 527 | 655 | 527 | 408 |
| Women’s Education - No schooling/missing | 66.6% | 55.0% | 54.5% | 56.4% |
| Women’s Education - Primary 1 | 11.2% | 23.8% | 22.2% | 9.1% |
| Women’s Education - Primary 2 | 11.2% | 13.9% | 15.9% | 15.2% |
| Women’s Education - Secondary 1, 2, university | 4.9% | 5.0% | 5.7% | 7.8% |
| Women’s Education - Other | 6.1% | 2.3% | 1.7% | 1.5% |
| Agroecology highland | 41.0% | 0.0% | 33.4% | 0.0% |
| Agroecology lowland | 19.2% | 49.2% | 0.0% | 24.5% |
| Agroecology midland | 39.8% | 50.8% | 66.6% | 75.5% |
| Access to Grid Electricity | 15.7% | 30.5% | 14.4% | 33.8% |
| Access to Improved Sanitation | 40.2% | 21.6% | 48.0% | 9.1% |
| Access to Improved Water | 78.2% | 82.7% | 84.9% | 89.9% |
| Women’s Dietary Diversity, median (IQR) | 3.0 (2.0, 3.0) | 3.0 (2.0, 4.0) | 2.0 (2.0, 3.0) | 3.0 (2.0, 3.0) |
| Minimum Dietary Diversity - Woman | 3.2% | 11.8% | 3.0% | 1.5% |
| Percent fasted yesterday | 29.8% | 1.4% | 1.3% | 25.7% |
| Market Diversity in November 2017, median (IQR) ^†^ | 2.0 (2.0, 4.0) | 5.0 (1.0, 7.0) | 5.0 (4.0, 8.0) | 7.0 (2.0, 9.0) |
| Total Food Crop Diversity (year), median (IQR) | 1.0 (1.0, 2.0) | 1.0 (1.0, 2.0) | 2.0 (1.0, 2.0) | 1.0 (1.0, 2.0) |
| Meher Food Crop Diversity, median (IQR) ^‡^ | 1.0 (1.0, 2.0) | 1.0 (1.0, 2.0) | 2.0 (1.0, 2.0) | 1.0 (1.0, 1.0) |
| Belg Food Crop Diversity, median (IQR) ^‡^ | 0.0 (0.0, 0.0) | 0.0 (0.0, 0.0) | 1.0 (1.0, 2.0) | 0.0 (0.0, 0.0) |
| Livestock Diversity, median (IQR) ^§^ | 3.0 (2.0, 4.0) | 3.0 (2.0, 4.0) | 3.0 (2.0, 4.0) | 3.0 (2.0, 4.0) |

† Percent with low market food diversity (less than 5 food groups); Refers to November 2016 market food diversity.

‡ Only out of 7 food groups because it does not include dairy, eggs, or meat food groups. Numbers represent among those who planted crops.

§ Among those who own specific livestock.

**Supplemental Table 2: Traditional classification of land uses and livestock systems in Ethiopia. Adapted from Table 5, Kocho and Geta (2011).**

|  | **Lowland** | **Midland** | **Highland** |
| --- | --- | --- | --- |
| Altitude (meters) | <1500 | 1500-2300 | >2300 |
| Rainfall (mm/year) | <800 | 800-1200 | >1200mm |
| Average annual temperature (^0^C) | >20 | 16-20 | <16 |
| Length of growing periods (days) | <120 | 120-240 | >240 |
| Moisture regimes | Arid to semi-arid | Sub-moist to moist | Sub-humid to per-humid |
| Total land area (million hectares) | 57.5 | 50.2 | 22.9 |

**Supplement Table 3: Coefficients for three models presented in Figure 3.**

|  | Bivariate | Multivariate no interaction | Multivariate with interaction |
| --- | --- | --- | --- |
| Market Diversity in November 2017 | -0.0233 | -0.00311 | -0.0308 |
|  | [-0.0667,0.0201] | [-0.0348,0.0286] | [-0.0841,0.0226] |
| Meher Food Crop Diversity | 0.127^*^ | 0.0959^*^ | 0.0877^*^ |
|  | [0.0635,0.191] | [0.0287,0.163] | [0.0204,0.155] |
| Belg Food Crop Diversity | 0.0536 | 0.0219 | 0.0201 |
|  | [-0.0226,0.130] | [-0.0592,0.103] | [-0.0607,0.101] |
| Amhara | Reference | Reference | Reference |
| Oromia | 0.431^*^ | 0.121 | -0.05 |
|  | [0.180,0.681] | [-0.133,0.374] | [-0.349,0.249] |
| SNNPR | -0.369^*^ | -0.407^*^ | -0.676^*^ |
|  | [-0.631,-0.107] | [-0.665,-0.148] | [-0.967,-0.385] |
| Tigray | -0.138 | -0.222 | -0.361^*^ |
|  | [-0.417,0.140] | [-0.500,0.0553] | [-0.635,-0.0873] |
| No schooling/missing | Reference | Reference | Reference |
| Primary 1 | 0.106^+^ | 0.0677 | 0.0684 |
|  | [-0.00950,0.221] | [-0.0471,0.182] | [-0.0463,0.183] |
| Primary 2 | 0.0509 | 0.025 | 0.0228 |
|  | [-0.0797,0.181] | [-0.110,0.160] | [-0.112,0.158] |
| Secondary 1, 2, university | 0.201^*^ | 0.187^+^ | 0.182^+^ |
|  | [0.0109,0.390] | [-0.0136,0.388] | [-0.0191,0.383] |
| Other (religious school, adult literacy) | -0.0736 | -0.128 | -0.132 |
|  | [-0.334,0.187] | [-0.384,0.128] | [-0.388,0.124] |
| Livestock Diversity | 0.106^*^ | 0.0949^*^ | 0.0954^*^ |
|  | [0.0678,0.144] | [0.0550,0.135] | [0.0556,0.135] |
| Cash crop | 0.260^*^ | 0.200^*^ | 0.214^*^ |
|  | [0.122,0.397] | [0.0617,0.337] | [0.0757,0.352] |
| Woman age | -0.00612^*^ | -0.00524 | -0.00532 |
|  | [-0.0117,-0.000524] | [-0.0120,0.00148] | [-0.0120,0.00139] |
| Total number of household members | 0.0181^+^ | 0.00525 | 0.004 |
|  | [-0.00297,0.0392] | [-0.0172,0.0277] | [-0.0184,0.0264] |
| Improved Sanitation | -0.215^*^ | -0.178^*^ | -0.180^*^ |
|  | [-0.313,-0.117] | [-0.276,-0.0804] | [-0.278,-0.0819] |
| Improved Water | 0.0362 | 0.0286 | 0.0243 |
|  | [-0.0922,0.165] | [-0.0958,0.153] | [-0.0996,0.148] |
| Lowland (agroecology) | Reference | Reference | Reference |
| Midland | -0.411^*^ | -0.263^*^ | -0.294 |
|  | [-0.657,-0.166] | [-0.482,-0.0436] | [-0.715,0.127] |
| Highland | -0.764^*^ | -0.485^*^ | -1.274^*^ |
|  | [-1.078,-0.449] | [-0.788,-0.181] | [-1.863,-0.684] |
| Wealth quintile 1 | Reference | Reference | Reference |
| Wealth quintile 2 | -0.0159 | -0.0336 | -0.0313 |
|  | [-0.155,0.123] | [-0.172,0.105] | [-0.169,0.107] |
| Wealth quintile 3 | 0.0471 | 0.017 | 0.0149 |
|  | [-0.0960,0.190] | [-0.126,0.160] | [-0.128,0.158] |
| Wealth quintile 4 | 0.113 | 0.0603 | 0.066 |
|  | [-0.0243,0.251] | [-0.0765,0.197] | [-0.0708,0.203] |
| Wealth quintile 5 | 0.277^*^ | 0.181^*^ | 0.180^*^ |
|  | [0.133,0.420] | [0.0364,0.325] | [0.0364,0.324] |
| Age of the Household Head | -0.00343 | -0.00384 | -0.00371 |
|  | [-0.00763,0.000766] | [-0.00854,0.000857] | [-0.00840,0.000986] |
| Farm size | 0.0258^*^ | 0.0148^*^ | 0.0154^*^ |
|  | [0.0164,0.0351] | [0.00494,0.0247] | [0.00561,0.0252] |
| Lowland x Market Diversity in November 2017 |  |  | Reference |
| Midland x Market Diversity in November 2017 |  |  | 0.0169 |
|  |  |  | [-0.0590,0.0928] |
| Highland x Market Diversity in November 2017 |  |  | 0.182^*^ |
|  |  |  | [0.0700,0.293] |

| 95% confidence intervals in brackets |
| --- |
| ^+^ *p* < 0.10, ^*^ *p* < 0.05 |
